# Clustered pattern projection for EEG dementia classification: evaluating the reliability of disorder patterns

**DOI:** 10.3389/fnins.2026.1871265

**Published:** 2026-07-30

**Authors:** Hunseok Kang, Jacob Kang, Mustafa Zeki, Jong-Hyeon Seo

**Affiliations:** 1 College of Engineering and Technology, American University of the Middle East, Egaila, Kuwait; 2 Fischell Department of Bioengineering, University of Maryland, College Park, MD, United States; 3 School of Basic Sciences, Hanbat National University, Daejeon, Republic of Korea

**Keywords:** Alzheimer's disease, clustered pattern projection, dynamic mode decomposition, electroencephalography (EEG), frontotemporal dementia, leave-one-subject-out cross-validation, margin-based reliability

## Abstract

**Introduction:**

Electroencephalography (EEG)-based classification of Alzheimer's disease (AD) and frontotemporal dementia (FTD) relative to cognitively normal (CN) controls is commonly interpreted through stable disease-related patterns. However, classification-relevant EEG responses in computational models may also appear fragmented or weakly preserved, making subject-level reliability difficult to assess. This study investigated whether recurrent disorder-like EEG patterns can provide exploratory evidence of subject-wise discriminative organization in dementia classification.

**Methods:**

We analyzed a publicly available resting-state EEG dataset including AD, FTD, and CN subjects. Clustered Pattern Projection (CPP) was applied to Dynamic Mode Decomposition (DMD)-based epoch descriptors to construct prototype-based EEG representations. Classification was performed using a linear support vector machine under a nested leave-one-subject-out cross-validation (LOSO-CV) framework. Subject-level reliability was further examined using margin-based analysis.

**Results:**

CPP showed competitive subject-level performance, particularly in the FTD vs. CN classification task, a setting in which resting-state discriminative patterns are often less consistently preserved than in AD. In addition, task-dependent subject-level margin patterns were observed under strict subject-wise validation, suggesting that margin analysis may provide a useful exploratory tool for evaluating the reliability of learned EEG representations.

**Discussion:**

These findings suggest that EEG generalization in dementia classification should not be interpreted only through preserved canonical biomarkers. Instead, recurrent disorder-like patterns may contribute to computationally detectable decision structure, and CPP provides a framework for examining such patterns under strict subject-wise validation.

## 1 Introduction

Dementia is a progressive neurodegenerative syndrome that impairs memory, cognition, and everyday functioning, and it represents a substantial global health burden. In 2021, approximately 57 million people were living with dementia worldwide, and nearly 10 million new cases occurred annually (World Health Organization, 2025). Alzheimer's disease (AD) is the most common dementia subtype and is estimated to contribute to 60%–70% of all dementia cases (Kamatham et al., 2024; World Health Organization, 2025). Among neurodegenerative dementias, frontotemporal dementia (FTD) is of particular clinical importance because its symptoms can partially overlap with those of AD, complicating differential diagnosis in practice, especially in heterogeneous or early-stage presentations (Ma et al., 2024a; Mlinarič et al., 2026; Pérez-Millan et al., 2024). In this context, electroencephalography (EEG) has attracted increasing attention as a non-invasive, accessible, and relatively low-cost modality for dementia assessment (Ehteshamzad, 2024; Yıldız and Zan, 2026). Accordingly, EEG-based classification of AD and FTD has been actively investigated using both conventional machine learning and recent deep-learning frameworks (Aviles et al., 2024; Mlinarič et al., 2026; Yıldız and Zan, 2026). Despite notable progress in convolutional and transformer-based approaches, practical EEG studies still frequently rely on conventional classifiers because available dementia datasets are often limited in size, methodologically heterogeneous, and difficult to generalize across cohorts (Aviles et al., 2024; Ehteshamzad, 2024; Shaikh et al., 2025).

Most existing resting-state EEG studies in dementia have been built on the premise that disease-related neural patterns are sufficiently stable across subjects to be captured by summary representations of spontaneous activity. Under eyes-closed (EC) conditions, this premise is often well justified in AD, where low-frequency slowing, reduced complexity, and decreased synchrony have been repeatedly reported and effectively characterized using spectral, entropy-based, connectivity, or Riemannian features (Cassani et al., 2018; Jeong, 2004; Rossini et al., 2020). As summarized in Table 1, EC-based dementia EEG studies have evaluated a broad range of feature representations, including entropy- and power-spectrum-based measures, recurrence-complexity features, mutual-information features, functional-connectivity measures, and Riemannian classifiers. These studies show that EC resting-state EEG can provide meaningful subject-level discriminative information for both AD and FTD. However, the reported performance varies substantially across diagnostic tasks and feature representations. In particular, disease–control classification is generally more tractable than AD–FTD discrimination, and FTD-related performance is often less stable, likely because low-frequency abnormalities are weaker, less consistent, or more heterogeneous across subjects; even so, useful discriminative information can still be partially recovered when the analysis is restricted to specific channels, connectivity measures, or frequency ranges (Mlinarič et al., 2026; Nishida et al., 2011; Zheng et al., 2025). By contrast, under eyes-open (EO) or stimulation-based conditions, AD has been associated with more degraded and unstable response patterns, and EEG studies in these settings remain relatively limited compared with the EC literature (de Waal et al., 2012; Kang et al., 2025).

**Table 1:** Overview of previous works on EC and EO EEG-based classification.

Study	Feature/validation	Problem	Classifier	Acc.	Sens.	Spec.	F1	AUC
Eyes closed								
Zandbagleh et al. (2024)	Multiscale dispersion entropy, PSD	AD/CN	LR	–	77.78	79.31	–	85.00
		FTD/CN	LR	–	56.52	79.31	–	67.00
		AD/FTD	LR	–	75.00	69.57	–	62.00
Ma et al. (2024b)	Mutual information, age, and sex	AD/CN	SVM	76.90	–	72.00	75.40	–
		FTD/CN	SVM	90.40	–	87.90	90.20	–
		AD/FTD	SVM	91.50	–	86.70	91.30	–
Zheng et al. (2024)	Recurrence complexity, hurst exponent, and rate gradient	AD/CN	SVM	87.69	–	75.86	–	–
		FTD/CN	SVM	82.69	–	89.66	–	–
		AD/FTD	SVM	72.88	–	39.13	–	–
		AD+FTD/CN	SVM	86.36	–	72.41	–	–
Mlinarič et al. (2026)	Multiple FC measures	AD/CN	Stacked ensemble	73.85	69.44	79.31	74.63	81.80
		FTD/CN	Stacked ensemble	71.15	69.57	72.41	68.09	71.36
		AD/FTD	Stacked ensemble	69.49	73.91	66.67	65.38	65.10
Mlinarič et al. (2026)	α-CSD, α-cross-covariance, δ-cross-correlation	AD/CN	FgMDM	84.62	77.78	93.10	84.85	88.70
		FTD/CN	FgMDM	82.69	73.91	89.66	79.07	86.36
		AD/FTD	FgMDM	64.41	52.21	72.22	53.33	70.65
Current study	DMD modes	AD/CN	DMD-CPP	76.70	76.19	77.34	76.53	79.48
		FTD/CN	DMD-CPP	85.44	82.61	87.69	85.19	86.42
		AD/FTD	DMD-CPP	67.07	78.57	49.07	64.07	62.25
Eyes open								
Ntetska et al. (2025)	PSD LNSO	AD/CN	SVM	62.50	–	47.20	66.80	–
		FTD/CN	LightGBM	71.00	–	83.30	67.30	–
Kang et al. (2025)	DMD modes LNSO	AD/CN	3D-CNN	74.23	–	–	73.83	–
		FTD/CN	3D-CNN	77.06	–	–	76.50	–
Seo et al. (2026)	DMD modes LOSO	AD/CN	DMD-CPP	84.75	88.24	80.00	86.96	–
		FTD/CN	DMD-CPP	83.33	77.27	88.46	80.95	–
		AD/FTD	DMD-CPP	68.52	79.41	50.00	76.06	–
Current study	DMD modes LOSO-CV	AD/CN	DMD-CPP	83.92	84.03	83.78	85.47	–
		FTD/CN	DMD-CPP	79.88	73.38	85.19	76.61	–
		AD/FTD	DMD-CPP	68.03	79.32	50.65	75.05	–

For EC studies listed here, LOSO-CV was used unless otherwise noted. Current-study values are from multi-seed LOSO-CV results; AUC is reported as the mean across seeds. Missing values are denoted by “–.” AUC, area under the curve; CNN, convolutional neural network; CPP, clustered pattern projection; CSD, cross-spectral density; DMD, dynamic mode decomposition; FC, functional connectivity; FgMDM, Fisher's geodesic minimum distance to mean; CN, cognitively normal control; LOSO-CV, leave-one-subject-out cross-validation; LR, logistic regression; PCA, principal component analysis; PSD, power spectral density; SVM, support vector machine.

This task- and feature-dependent variability suggests that classification-relevant EEG information is not always expressed as a globally stable abnormality across all channels or frequency components. Instead, some information may be concentrated in selected feature subspaces. For example, Ma et al. (2024b) showed that electrode-pair communication features can contribute differently to AD, FTD, and CN classification, and that feature-importance-based refinement can improve subtype discrimination. Such findings imply that signal components that appear weak, irregular, or even detrimental in a global EEG summary may still contain reproducible diagnostic structure when viewed through an appropriate task-specific representation. In this study, we use the term “disorder pattern” in this restricted sense: an apparently weak, fragmented, or non-canonical EEG configuration that is not treated as arbitrary noise, but is considered potentially meaningful only when it recurs in a task-relevant computational feature space and contributes to subject-level discrimination. These observations raise an important question: are apparently weak or disordered EEG responses merely detrimental to classification, or can they themselves contain reproducible subject-level decision structure?

A previous EO-based study (Seo et al., 2026) showed that degraded or unstable AD-related EO responses may still contain classification-relevant structure when represented through Clustered Pattern Projection (CPP)-based prototype projection. This finding suggested that disease-relevant information may not reside only in well-preserved canonical oscillatory patterns, but also in recurrent breakdown-like patterns that reappear across subjects and epochs. Table 1 also illustrates that EO or stimulation-based EEG classification remains less extensively characterized than EC resting-state classification, with fewer directly comparable subject-level reports across AD, FTD, and CN tasks. Thus, EO findings are useful for motivating the possibility of disorder-pattern learning, whereas EC studies provide a more established benchmark for testing whether such patterns can generalize under strict subject-wise validation. In other words, response configurations previously regarded as unstable or unfavorable may still function as class-associated anchors in a prototype-based representation space. Motivated by this observation, we hypothesize that EEG conditions traditionally regarded as unfavorable for conventional classification may nevertheless contain recurrent task-relevant structure detectable by computational representation learning. In the present study, we test this hypothesis in a representative difficult setting, namely FTD under EC, where resting-state discriminative patterns are often weaker or less consistently preserved than in AD, and where disease–control classification has been reported to be less accurate than for AD in recent EEG studies (Mlinarič et al., 2026; Nishida et al., 2011; Zheng et al., 2025). If this hypothesis is correct, CPP should not only provide competitive classification performance but also yield more reliable decision geometry, reflected in clearer subject-level margin separation under stricter validation.

Although many prior EEG studies have reported high dementia-classification accuracies, sometimes approaching or exceeding 90%, the extent to which epoch-level predictions were translated into reliable subject-level decisions has often remained unclear (Mlinarič et al., 2026; Modir et al., 2023). This issue is particularly important in EEG because subject-specific structure can be strong, and if epochs from the same participant appear in both training and test sets, the classifier may exploit subject identity rather than disease-related patterns, thereby yielding overly optimistic estimates of generalization (Brookshire et al., 2024; Del Pup et al., 2025; Kang et al., 2025). Subject-wise schemes, such as leave-one-subject-out (LOSO), therefore provide a necessary baseline for evaluating cross-subject performance, but they are not sufficient by themselves to guarantee that the learned representation reflects a genuinely recurrent subject-level decision pattern, especially in small and heterogeneous EEG datasets where non-stationarity and inter-subject variability remain substantial (Del Pup et al., 2025; Modir et al., 2023). For this reason, in our previous study, we complemented LOSO-based evaluation with margin analysis and showed that false positives and true positives occupied systematically different regions of the decision space, supporting the interpretation that the representative CPP clusters captured disorder-related structure rather than merely idiosyncratic subject patterns (Seo et al., 2026). In the present work, we extend this reliability-oriented perspective by explicitly examining whether competitive performance under strict subject-wise validation is accompanied by subject-level margin separation, thereby providing additional exploratory evidence that the discovered CPP prototypes reflect recurrent subject-level decision structure.

## 2 Materials and methods

### 2.1 Dataset and ethical considerations

This study used the publicly available OpenNeuro dataset ds004504, which contains resting-state, eyes-closed EEG recordings from subjects with AD, FTD, and CN (Miltiadous et al., 2023a,b). The dataset comprises 88 participants in total, including 36 subjects with AD, 23 with FTD, and 29 CN. Demographic and clinical characteristics of the cohort are summarized in Table 2, while the main EEG acquisition and dataset specifications are listed in Table 3.

**Table 2:** Demographic and clinical summary.

Variable	AD (*n* = 36)	FTD (*n* = 23)	CN (*n* = 29)
Age (years)	66.4 ± 7.9	63.6 ± 8.2	67.9 ± 5.4
Female, *n* (%)	24 (67%)	9 (39%)	11 (38%)
MMSE	17.75 ± 4.5	22.17 ± 2.6	30.0 ± 0.0

Values are presented as mean ± standard deviation, except sex. AD, Alzheimer's disease; FTD, frontotemporal dementia; CN, cognitively normal controls; MMSE, Mini-Mental State Examination. Group-level statistical comparisons for this cohort were reported previously in Mlinarič et al. (2026).

**Table 3:** Summary of the publicly available EEG dataset.

Item	Description
Dataset	OpenNeuro ds004504
Source paper	Miltiadous et al. (2023b)
Recording condition	Resting state, eyes closed
Groups	AD, FTD, CN
Number of subjects	88 total (36 AD, 23 FTD, 29 CN)
EEG montage	19 scalp electrodes (+2 mastoid electrodes)
Electrode system	International 10–20 system
Sampling rate	500 Hz
Shared data version used	publicly released preprocessed EEG
Preprocessing in shared dataset	0.5–45 Hz band-pass filtering; ASR; ICA artifact removal; common-average rereferencing; automated bad trial/channel handling

The recordings were obtained during resting state with eyes closed using 19 scalp electrodes placed according to the international 10–20 system, together with two mastoid electrodes, at a sampling rate of 500 Hz. Recording duration varied across subjects and diagnostic groups, but all data were acquired under the same resting-state protocol. Ethical approval did not apply to the present study because the authors collected no new data. All EEG recordings used here were fully anonymized and obtained from an existing public dataset released for research purposes. The original data collection protocol was approved by the Scientific and Ethics Committee of AHEPA University Hospital, Aristotle University of Thessaloniki.

In this work, we analyzed the preprocessed EEG signals distributed by the dataset authors. According to the dataset description, the released recordings had already undergone band-pass filtering, artifact attenuation and rejection, rereferencing, and epoch-level cleaning. Because the shared preprocessed EEG was used as provided, no additional preprocessing was performed in the present study before feature extraction (Miltiadous et al., 2023b; Mlinarič et al., 2026).

In addition, we directly examined the recording durations available in the dataset. Because this dataset consists of eyes-closed resting-state EEG with no task events or stimulus onsets, there were no epoch-defining markers; the entire continuous recording for each subject constituted the usable data. Accordingly, we used the full available recording duration for every subject without applying any event-based trimming.

As summarized in Table 4, mean recording lengths were broadly comparable across groups (AD: 808.3 ± 164.5 s; CN: 830.4 ± 66.2 s; FTD: 720.4 ± 141.7 s), though notable variability exists, particularly within the AD group (range: 306–1,280 s) and the FTD group (range: 478–1,014 s). The CN group showed the most consistent durations (range: 748–988 s). The FTD group had a somewhat shorter mean recording length compared to AD and CN, and the AD group exhibited the highest within-group variance.

**Table 4:** Usable EEG recording lengths by diagnostic group.

Group	Usable *N*	Mean ±SD (s)	Min (s)	Max (s)
AD	36 / 36	808.3 ± 164.5	306	1,280
CN	29 / 29	830.4 ± 66.2	748	988
FTD	23 / 23	720.4 ± 141.7	478	1,014

We acknowledge this as a limitation: unequal and variable recording durations across groups could introduce bias, as subjects with longer recordings contribute more 2-s segments to DMD estimation, potentially yielding more stable spectral descriptors. Although our epoch-based pipeline mitigates this to some degree by sampling a fixed number of epochs per subject regardless of total recording length, the underlying variability in data quantity remains a confound that warrants caution in interpreting group-level differences.

### 2.2 Feature extraction

This subsection describes the construction of fixed-size, mode-based EEG descriptors from resting-state recordings. Unlike previous EO stimulus-based settings (Seo et al., 2026), the present data consist of continuous EC resting-state EEG without an external event structure. Accordingly, each subject's usable recording was divided into 10 consecutive epochs covering the full available duration, with little or no overlap between neighboring epochs. Each epoch was then partitioned into non-overlapping 2-s segments, and Dynamic Mode Decomposition (DMD) was applied to every segment to obtain rectified mode matrices together with their associated eigenfrequencies. Within each epoch, the segment-wise mode-magnitude matrices were aggregated over time and summarized by temporal statistics, yielding fixed-length representations that preserve sensor-space structure and are suitable for subsequent pattern learning and classification.

#### 2.2.1 Dynamic mode decomposition

DMD provides a modal representation of multichannel time series in terms of coherent spatio-temporal components and their associated eigenvalues (Schmid, 2010). Because EEG recordings typically contain far fewer channels than temporal samples, we use an extended (stacked) DMD formulation based on concatenated time-delay observations.

In the actual computation, DMD is first formulated in discrete time. Let ${\text{x}}_{k}\in {\mathbb{R}}^{M}$ denote the multichannel EEG sample at time index *k*. In the stacked formulation, consecutive delayed observations are concatenated to form an augmented state vector. DMD then estimates a finite-dimensional time-advance operator that maps the stacked state at one time step to the stacked state at the next time step. That is, the discrete evolution is approximated as **y**_*k*+1_≈**Ay**_*k*_, where **y**_*k*_ is the stacked observation vector and **A** is the reduced Koopman, or DMD, time-advance operator estimated from the paired snapshot matrices. The eigenvalues of this discrete operator describe the one-step temporal evolution of the stacked EEG observations. These discrete-time eigenvalues are then converted into continuous-time eigenvalues, which are used to express the modal expansion below.

The resulting continuous-time signal model is written as:


$$\begin{array}{l} \text{x}\left(t\right)\approx \underset{j}{\sum }c_{j}\phi_{j}e^{\omega_{j}t}, \end{array}$$ (1)

where **x**(*t*) is the multichannel EEG signal with *M* channels, **ϕ**_*j*_ denotes the *j*th DMD mode, ω_*j*_ is the corresponding continuous-time eigenvalue converted from the discrete-time DMD eigenvalue, and *c*_*j*_ is the modal amplitude.

In the stacked DMD formulation, the construction of the augmented state and the reduced-order approximation are controlled by two parameters: the stacking size *S*, which determines the number of time-delay embeddings, and the truncation rank *R*, which specifies the number of retained modes after dimensionality reduction. In addition, each EEG recording is partitioned into segments and epochs characterized by parameters such as the number of samples per segment *N*. The definitions and values of all symbols used in this study are summarized in Table 5.

**Table 5:** Summary of symbols and parameters used in the DMD-based feature construction.

Symbol	Meaning
*M* = 19	Number of EEG channels
*N* = 1,000	Number of samples per segment (2-s at 500Hz)
*S* = 48	DMD stack size (time-delay embedding length)
*R* = 100	Truncation rank (number of retained modes)
*P* = 50	Mode-axis resolution for descriptor construction

In this study, DMD was applied to each 2-s segment, and the extracted mode information was used to construct epoch-level descriptors. Further implementation details of the augmented DMD procedure are given in Appendix A1 of Seo et al. (2026).

#### 2.2.2 DMD configuration and post-processing

For each subject, the usable resting-state EEG recording was divided into 10 consecutive epochs spanning the full available duration. Each epoch was then subdivided into non-overlapping 2-s segments. DMD was performed once for each non-overlapping 2-s segment, and the resulting mode matrix was used for subsequent epoch-level aggregation. To reduce sensor-uniform contributions and to mitigate artifacts such as volume-conduction-related inflation and machine-harmonic contamination, we applied a simple postprocessing step to each DMD mode. Specifically, for each mode vector, we subtracted the mean of its channel-wise absolute values and used the resulting magnitude-adjusted representation in all subsequent analyses. Thus, the present study relies on magnitude-based rectified DMD modes. Additional implementation details of this postprocessing procedure are provided in Appendix A2 of Seo et al. (2026).

Although the DMD modes are complex-valued and their phases may contain relative timing information across channels, the present study used magnitude-based rectified modes to focus on the spatial participation strength of each dynamic component. This choice was made because the aim of the study was not to estimate phase-based functional synchronization, but to construct stable channel-by-mode descriptors suitable for prototype-based classification under subject-wise validation. Magnitude-based descriptors also reduce sensitivity to inter-subject and epoch-level phase variability and yield non-negative mode images that can be consistently compared using cosine similarity. Therefore, phase information was not interpreted as irrelevant, but was outside the scope of the present representation.

#### 2.2.3 Clustered pattern projection (CPP)

In this study, we employ the Clustered Pattern Projection (CPP) framework introduced by Seo et al. (2026). CPP consists of two main stages: (i) construction of epoch-level descriptors from DMD-based representations, and (ii) learning of representative pattern dictionaries followed by projection-based feature extraction.

In the first stage, each epoch is represented by a fixed-size descriptor that summarizes the spatial distribution of DMD modes across EEG channels. Specifically, DMD is applied to each 2-s segment, and only modes whose oscillatory frequencies, computed from the corresponding continuous-time eigenvalues ω_*j*_ in Equation 1, fall within the 4–40 Hz range are retained. The detailed conversion from DMD eigenvalues to modal frequencies follows Appendix A1 of Seo et al. (2026). The retained mode magnitudes are organized into a non-negative mode-image and aggregated within each epoch to form a descriptor:


$$\hat{\text{D}}\in {\left[0,1\right]}^{M\times P},$$

where *M* denotes the number of EEG channels and *P* is the normalized mode dimension. The descriptor is then vectorized as:


$$\begin{array}{l} \text{z}=\text{vec}\left(\hat{\text{D}}\right)\in {\mathbb{R}}^{MP}, \end{array}$$ (2)

yielding a fixed-length representation for each epoch. To obtain fixed-size descriptors, the retained mode dimension is normalized onto a predefined grid with resolution *P*. In this study, we use *P* = 50, as summarized in Table 5.

In the second stage, representative patterns are learned from collections of descriptors. Given a subset of descriptors indexed by I, the corresponding design matrix is defined as:


$$\begin{array}{l} {\text{Z}}_{I}=\left[{\text{z}}_{n}:n\in I\right]. \end{array}$$ (3)

Hierarchical divisive clustering with cosine dissimilarity is applied to ${\text{Z}}_{I}$ to identify coherent groups of descriptors.

The clustering process is governed by three hyperparameters: the cluster size threshold τ_sup_, the minimum cluster size *n*_min_, and the stopping height *h*_stop_. The parameter τ_sup_ determines whether a subset is sufficiently large to be further partitioned, *n*_min_ enforces a lower bound on the size of resulting clusters, and *h*_stop_ controls the minimum dendrogram height required to perform a split. These parameters define the granularity and stability of the learned clustering structure.

From each cluster, a representative descriptor (medoid) is selected to construct the dictionary:


$$\begin{array}{l} \text{B}=\left[{\text{b}}_{1},\ldots ,{\text{b}}_{K}\right]\in {\mathbb{R}}^{MP\times K}. \end{array}$$ (4)

Finally, each descriptor **z** is mapped to a feature vector by computing cosine similarity with the learned dictionary atoms,


$$\begin{array}{l} f_{k}\left(\text{z}\right)=\frac{{\text{z}}^{⊤}{\text{b}}_{k}}{||\text{z}|{|}_{2}||{\text{b}}_{k}|{|}_{2}},\;k=1,\ldots ,K, \end{array}$$ (5)

resulting in a *K*-dimensional feature representation that encodes the relationship between each sample and the learned pattern prototypes.

The CPP framework used in this study follows our previous formulation; for full implementation details, including descriptor construction and clustering criteria, we refer to Section 2.2 of Seo et al. (2026).

#### 2.2.4 Classification procedure

We consider a binary classification task τ with class set $Y_{\tau }=\left\{c_{1},c_{2}\right\}$. For each epoch, the vectorized descriptor ${\text{z}}_{n}\in {\mathbb{R}}^{MP}$ is obtained as defined in Equation 2. The design matrices for the training and test splits are obtained as instances of Equation 3 by taking the index set I to correspond to the training and test samples, respectively, and are denoted by **Z**^train^ and **Z**^test^.

Using the training data only, class-specific dictionaries ${\text{B}}_{c}\in {\mathbb{R}}^{MP\times K_{c}}$ in Equation 4 are constructed for each class $c\in Y_{\tau }$ via the clustering procedure. Each descriptor **z** is mapped to a feature vector by projecting it onto the class-specific dictionary **B**_*c*_ using cosine similarity in Equation 5, resulting in class-specific feature blocks ${\text{F}}_{c}^{A}$ for each split A∈{train,test}. These are concatenated to form the final representation:


$${\text{H}}_{\tau }^{A}=\left[{\text{F}}_{c_{1}}^{A}\;{\text{F}}_{c_{2}}^{A}\right],\;A\in \left\{\text{train},\text{test}\right\}.$$

To ensure comparability across subjects, each feature dimension is standardized using the mean and standard deviation computed from the training split. A linear SVM is then trained on the normalized training features and applied to the test features for prediction. In this study, no class weights were applied in the SVM cost function. This choice was made to assess whether the proposed CPP representation, based on class-specific medoid dictionaries and cosine-projection features, could improve subject-level separability without an additional cost-sensitive correction at the classifier stage. Evaluation metrics are reported in accordance with the protocol described in the next subsection.

A linear SVM was selected deliberately rather than a deep-learning model or a more flexible non-linear classifier. This choice was motivated by three considerations. First, the number of subjects in the dataset was limited, especially for the FTD group, whereas the DMD-CPP feature representation remained relatively high-dimensional. In this small-sample, high-dimensional setting, a linear classifier yields a more conservative decision model and reduces the risk that classification performance is driven by overly flexible decision boundaries (Hsu et al., 2003). Second, the main objective of this study was not to maximize classification accuracy using a complex classifier, but to examine whether the CPP representation itself produces recurrent class-associated prototype structures that support subject-level discrimination. Third, the decision-margin analysis used in this study requires an interpretable signed distance to a decision boundary. A linear SVM provides a direct margin with respect to a single separating hyperplane, allowing subject-level confidence magnitude, within-subject dispersion, and sign consistency to be analyzed transparently. In contrast, non-linear kernels or deep-learning models could introduce model-specific non-linear interactions that make it more difficult to determine whether the observed decision structure arises from recurrent CPP medoid projections or from the classifier itself.

#### 2.2.5 Nested LOSO-CV evaluation

To evaluate subject-level generalization, we adopted a leave-one-subject-out cross-validation (LOSO-CV) protocol, in which all epochs from one subject were held out as the test set while the remaining subjects were used for training (Varoquaux et al., 2017). Such subject-independent evaluation is essential in EEG analysis because of substantial inter-subject variability and the risk of data leakage when epochs from the same subject appear in both training and test sets (Roy et al., 2019).

Because the final classification target was the subject rather than the individual epoch, epoch-level outputs were aggregated at the subject level in both the inner model-selection loop and the outer evaluation loop. For each subject, the 10 epoch-level predictions were converted into a single subject-level decision by majority voting. A subject was assigned to the positive class when at least half of its epochs were predicted as positive; otherwise, it was assigned to the negative class. The same aggregation rule was used whenever epoch-level predictions were converted into subject-level decisions.

A nested LOSO-CV scheme was used to separate model selection from final performance evaluation (Varoquaux et al., 2017). In each outer LOSO fold, one subject was held out for testing, and all hyperparameter selection was performed using only the remaining training subjects. Within the inner loop, candidate model configurations were evaluated using inner-training and inner-validation subjects only. For the proposed clustering-based model, each candidate configuration corresponded to one triplet of clustering hyperparameters (τ_sup_, *n*_min_, *h*_stop_).

The inner-loop model-selection criterion was fixed to the area under the receiver operating characteristic curve (ROC-AUC). To compute this AUC, the 10 epoch-level decision margins of each inner-validation subject were summarized by their median, yielding one decision score per subject. These subject-level scores, together with the corresponding subject labels, were used to compute the inner-loop AUC with the positive class as the reference class. The candidate configuration with the highest AUC was selected for the corresponding outer fold. Therefore, the outer held-out subject was not used at any stage of hyperparameter selection.

Note that for each candidate configuration in the inner loop, all CPP model components were estimated using only the inner-training subjects. Specifically, class-specific CPP dictionaries were constructed by hierarchical clustering and medoid selection within the inner-training set, feature standardization parameters were computed from the inner-training features, and the linear SVM was trained only on the standardized inner-training features. The inner-validation subjects were used only to compute the validation AUC for hyperparameter selection.

After the optimal configuration was selected, the model was retrained using all outer-training subjects and then applied to the outer held-out subject. For the held-out subject, the 10 epoch-level predictions were aggregated into a single subject-level prediction using the same majority-voting rule. In parallel, the 10 epoch-level decision margins were summarized by their median to obtain one subject-level decision score for ROC-AUC computation. The outer held-out subject was used only for the final performance evaluation.

Performance metrics were computed at the subject level. Since each outer LOSO fold yields one held-out subject, metrics were not computed separately for individual folds. Instead, after completing the full outer LOSO cycle for a given random seed, the subject-level predictions and subject-level decision scores from all held-out subjects were collected.

The subject-level predictions and true labels were first used to construct a confusion matrix. From this confusion matrix, accuracy, sensitivity, and specificity were computed as conventional threshold-based measures to support comparison with previous EEG-based dementia-classification studies. In addition to these standard measures, we computed ROC-AUC, balanced accuracy, raw precision, macro-F1, and the Matthews correlation coefficient (MCC) to account for class imbalance and to more explicitly characterize false-positive behavior and overall confusion-matrix balance. ROC-AUC was computed using the subject-level decision scores and true labels. It was used as the primary metric for comparing the proposed DMD-CPP method with the DMD-PCA baseline, because it summarizes discriminative ability across decision thresholds and is less tied to a particular operating threshold. Balanced accuracy was computed as:


$$\text{Balanced Accuracy}=\frac{1}{2}\left(\text{Sensitivity}+\text{Specificity}\right),$$

which gives equal weight to the positive and negative classes. Precision was included to clarify the effect of false positives, particularly the frequency with which subjects from the negative class were incorrectly assigned to the positive diagnostic class. Macro-F1 was computed as the unweighted average of the class-wise F1-scores, thereby giving equal contribution to both classes regardless of their sample sizes. MCC was computed as:


$$\text{MCC}=\frac{\text{TP}\cdot \text{TN}-\text{FP}\cdot \text{FN}}{\sqrt{\left(\text{TP}+\text{FP}\right)\left(\text{TP}+\text{FN}\right)\left(\text{TN}+\text{FP}\right)\left(\text{TN}+\text{FN}\right)}},$$

where TP, FP, TN, and FN denote true-positive, false-positive, true-negative, and false-negative subject-level decisions, respectively. MCC was included because it evaluates all four entries of the confusion matrix symmetrically and is therefore appropriate for assessing classification performance under unequal class sizes (Matthews, 1975).

To account for variability due to the inner model-selection process, the complete nested LOSO-CV procedure was repeated using seven random seeds. For each seed, the full outer LOSO cycle produced one set of subject-level performance metrics. Final performance was reported as the mean and standard deviation of each metric across the seven repeated nested LOSO-CV runs.

To assess the discriminative power of the extracted features, we evaluated three binary discrimination problems under the nested LOSO-CV protocol: AD vs. CN, FTD vs. CN, and AD vs. FTD. The positive class was defined as AD for AD vs. CN, FTD for FTD vs. CN, and AD for AD vs. FTD.

The present study focused on three binary contrasts rather than joint multiclass AD/FTD/CN classification because the primary aim was to evaluate task-specific CPP representation geometry and binary decision-margin reliability for each disease-group comparison.

### 2.3 Decision-margin analysis

To assess model reliability beyond classification accuracy, we perform a decision-margin analysis based on the decision scores produced by the linear SVM. For each test epoch, the classifier outputs a signed margin *m*, defined as the distance to the separating hyperplane. The magnitude of the margin is commonly interpreted as a measure of prediction confidence, while its sign determines the predicted class.

In this study, margin analysis was also used to examine whether weak, fragmented, or non-canonical medoid patterns captured by CPP were associated with reproducible subject-level decision structure. Such medoids should not be interpreted as disorder-related patterns based on visual appearance alone. Rather, their relevance should be supported by the projection behavior of held-out subjects in the learned CPP feature space. Correctly classified subjects are expected to show stronger or more consistent margins, whereas misclassified subjects may show weaker, more dispersed, or less stable projections toward the learned medoid structure.

In the previous study (Seo et al., 2026), margin-based analysis was introduced as a tool to characterize the reliability and structure of learned representations. In the present work, we extend this framework to a repeated LOSO-CV setting, enabling more robust statistical evaluation of subject-level margin behavior.

#### 2.3.1 Subject-level margin descriptors

Because clinical decisions are made at the subject level, margin analysis is performed by first aggregating epoch-wise margins within each subject. For a given subject *s*, let ${\left\{m_{s,e}\right\}}_{e=1}^{E}$ denote the set of margins over its *E* epochs (in this study, *E* = 10). From this set, we compute three subject-level descriptors:

1. Confidence magnitude:

$$\text{media}{\text{n}}_{e}\left(\left|m_{s,e}\right|\right)$$

which summarizes the typical distance of the subject's samples from the decision boundary.

2. Within-subject dispersion:

$$\text{IQ}{\text{R}}_{e}\left(m_{s,e}\right)$$

which captures variability of margins across epochs.

3. Within-subject sign consistency:

$$\left|P_{e}\left(m_{s,e}>0\right)-0.5\right|$$

where *P*_*e*_(*m*_*s, e*_>0) denotes the proportion of epochs with positive margins. This measures directional stability across epochs.

Thus, each subject is represented by three scalar margin descriptors.

#### 2.3.2 Statistical analysis

The proposed framework constructs projection features anchored to disease-related break patterns learned during clustering. Under this design, subjects whose data consistently match the learned disease-related anchors are expected to exhibit distinct decision-margin behavior from subjects whose positive projections arise from spurious or unstable matches. Therefore, the primary reliability analysis focused on whether the subject-level margin descriptors differed between correctly identified patients (TP) and subjects incorrectly projected toward the patient class (FP). Comparisons between correctly identified controls (TN) and missed patients (FN) were analyzed as complementary evidence.

Reliability analysis was performed at the subject level using the repeated nested LOSO-CV outputs. For each random seed, the 10 epoch-level predictions of each held-out subject were aggregated into one subject-level prediction using the voting rule defined above. Based on the true subject label and this subject-level prediction, each subject was assigned to one of the four outcome categories: TP, FP, TN, or FN. The margin descriptors used in this analysis were the three subject-level descriptors defined in the previous subsection. Thus, for each seed and each subject, one outcome category and one set of subject-level margin descriptors were obtained. These seed-specific subject summaries were then used to compare the margin behavior across the TP, FP, TN, and FN groups.

For each descriptor, group differences were summarized using the median difference,


$$\Delta \text{med}=\text{median}\left(X_{A}\right)-\text{median}\left(X_{B}\right),$$

where *X*_*A*_ and *X*_*B*_ denote the descriptor values in the two outcome groups being compared. The primary comparison was TP vs. FP, and the complementary comparison was TN vs. FN. Uncertainty in the median difference was quantified using percentile bootstrap confidence intervals. Statistical significance was assessed using two-sided permutation tests with fixed group sizes, in which the seed-specific subject descriptor values were randomly reassigned between the two outcome groups. This procedure evaluates whether the observed median separation is larger than expected under the null hypothesis that outcome-group membership carries no systematic information about the descriptor.

Because repeated seed runs generate multiple summaries for the same subjects, these seed-specific subject summaries were interpreted as repeated evaluation outcomes rather than as fully independent biological subjects. Accordingly, this analysis was treated as an exploratory, stability-oriented non-parametric assessment of subject-level margin behavior across repeated nested LOSO-CV runs. All tests were conducted at a significance level of α = 0.05. In addition to median differences and permutation *p*-values, effect size was quantified using Cliff's δ, a non-parametric measure of stochastic dominance defined as the probability that a randomly selected value from one group exceeds a randomly selected value from the other group minus the reverse probability. Cliff's δ ranges from −1 to 1, with values near zero indicating substantial overlap between groups and larger absolute values indicating stronger separation (Cliff, 1993). Bootstrap confidence intervals were also computed for Cliff's δ.

### 2.4 Comparison algorithm: PCA-based mode features

To assess the contribution of the proposed clustering-based Stage 2 representation, we included a PCA-based comparison pipeline. Principal component analysis (PCA) is a standard linear dimensionality-reduction method that projects data onto an orthogonal subspace whose axes successively maximize the variance of the projected data (Jolliffe, 2002). This comparison follows the same general baseline strategy used in the previous study (Seo et al., 2026), but was re-evaluated here under the same nested LOSO-CV protocol as the proposed method. The PCA-based pipeline uses the same Stage 1 mode-based epoch descriptors as the proposed DMD-CPP framework, but replaces class-specific clustering and medoid-based dictionary construction with PCA-based linear projection. Thus, the comparison isolates the effect of the Stage 2 representation: disease-pattern-oriented clustering and prototype projection in the proposed method vs. global variance-preserving linear projection in the PCA baseline.

#### 2.4.1 Shared stage 1 mode-based representation

For each epoch, the same Stage 1 mode-based descriptor **z**∈ℝ^*MP*^ was constructed as described in Equation 6. Thus, both the proposed DMD-CPP method and the PCA-based comparison method start from identical DMD-derived epoch representations. This design isolates the effect of the Stage 2 feature projection method, namely clustering-based prototype projection vs. PCA-based linear dimensionality reduction.

For a given binary classification task τ with class set $Y_{\tau }=\left\{c_{1},c_{2}\right\}$, task-specific training and test design matrices were constructed using only subjects belonging to the two classes of interest. Let A∈{train,test} denote the data split under an outer LOSO fold, and let $I_{\tau }^{A}$ be the corresponding task-restricted subject index set. The design matrix was written as:


$$\begin{array}{l} {\text{Z}}_{\tau }^{A}=\left[{\text{z}}_{n}:n\in I_{\tau }^{A}\right], \end{array}$$ (6)

where each column corresponds to one epoch-level descriptor from a subject belonging to task τ.

#### 2.4.2 PCA-based projection and inner-loop model selection

Within each outer LOSO fold, PCA model selection was performed using only the outer-training subjects. Candidate PCA configurations were defined by the retained-variance threshold. Here, the retained-variance threshold determines the number of principal components to retain: principal components are ordered by explained variance, and the smallest number of components whose cumulative explained variance reaches the specified threshold is selected. Thus, this parameter controls the dimensionality of the PCA-projected feature space while preserving a prescribed proportion of the variance in the training data.

For each candidate retained-variance threshold, PCA was fitted only on the inner-training data. The corresponding inner-validation data were then projected using the PCA basis learned from the inner-training split and standardized using inner-training statistics. A linear SVM was used for classification in all PCA-based experiments. A linear SVM was used for classification in all PCA-based experiments, consistent with the proposed DMD-CPP pipeline. This choice was made not only for methodological consistency, but also because the PCA-projected mode-feature representation remained relatively high-dimensional, and linear SVMs are commonly effective in such settings (Hsu et al., 2003). Epoch-level outputs from the inner-validation subjects were aggregated at the subject level using the same voting rule as the proposed method, and the resulting subject-level scores were used to compute the inner-loop AUC.

### 2.5 Experimental setup

All postprocessing, feature extraction, and classification were implemented in Matlab 2023b. The experiments were conducted under a nested LOSO-CV protocol to ensure subject-independent evaluation and training-only model selection. For each binary classification task, model-selection hyperparameters were chosen within the inner loop using only the outer-training subjects, and the selected configuration was then evaluated on the outer held-out subject.

The DMD descriptor-construction parameters summarized in Table 5 were fixed before the nested LOSO-CV experiments and were not selected within the inner cross-validation loop. These values were chosen empirically through repeated preliminary experiments, following the previous DMD-CPP configuration (Seo et al., 2026), while considering classification performance, numerical stability, descriptor resolution, and computational cost. The retained 4–40 Hz range was used to focus on theta-to-gamma modal dynamics; delta-band components were excluded because, with 2-s segments, they mainly reflect slower long-term fluctuations and may be less suitable for local DMD mode characterization (Kang et al., 2025). The same parameters were used across all tasks, methods, folds, and random seeds to ensure a common descriptor space and fair comparison between DMD-CPP and DMD-PCA. A full sensitivity analysis of these DMD parameters was not performed in the present study and is left for future work.

Inner-loop model selection was based on AUC, as defined in the nested LOSO-CV procedure. The complete nested LOSO-CV experiment was repeated using seven random seeds for the three binary classification tasks: AD vs. CN, FTD vs. CN, and AD vs. FTD.

For the proposed DMD-CPP method, the inner-loop candidate configurations were defined by the clustering hyperparameters used in the Stage 2 dictionary construction. Specifically, the stopping height *h*_stop_, the minimum minority-cluster size *n*_min_, and the cluster support threshold τ_sup_ were searched over predefined grids. The basis type was fixed to medoid extraction, hierarchical clustering used complete linkage, and feature vectors were normalized to unit norm before cosine projection.

Thus, Stage 1 descriptor construction was applied independently to each subject's epochs using fixed parameters, whereas all data-dependent Stage 2 learning procedures were performed within the corresponding training split.

For the PCA-based comparison pipeline, the inner-loop candidate configurations were defined by the PCA retained-variance threshold. The retained-variance threshold determines the smallest number of principal components whose cumulative explained variance reaches the specified percentage. The classifier was fixed to a linear SVM for the PCA-based comparison, so that the baseline differed from the proposed method primarily in the Stage 2 feature projection strategy rather than in the classifier family.

The candidate configuration grids used in the inner loop are summarized in Table 6.

**Table 6:** Candidate configurations used for inner-loop model selection.

Method	Parameter	Candidate values/setting
DMD-CPP	Stopping height, *h*_stop_	{0.15, 0.20, 0.25, 0.30}
	Minimum minority-cluster size, *n*_min_	{4, 6, 8, 10, 12, 14}
	Cluster support threshold, τ_sup_	{3, 5, 7, 9, 11, 13, 15}
	Basis type	Medoid
	Linkage rule	Complete linkage
	Unit normalization	Enabled
PCA baseline	Retained-variance threshold	{80, 85, 90, 92, 95, 97, 99}%
	Classifier	Linear SVM
Both	Inner-loop selection metric	AUC
	Random seeds	{70, 85, 100, 115, 130, 145, 160}

## 3 Results

### 3.1 Seed-averaged ROC curves

**Figure 1:**
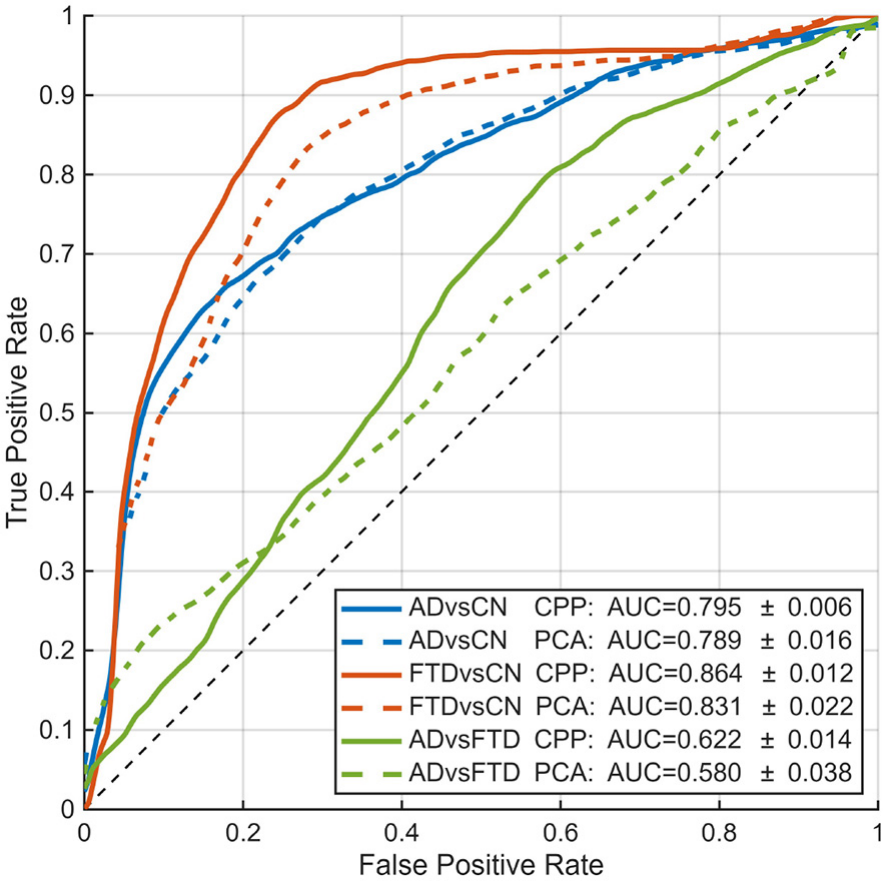
Seed-averaged ROC curves across classification tasks and methods. ROC curves are shown for DMD-CPP (solid) and DMD-PCA (dashed) across three tasks (AD vs. CN, FTD vs. CN, and AD vs. FTD). For each method and task, ROC curves were computed per seed under LOSO-CV validation and interpolated onto a common FPR grid, then averaged to obtain a representative curve. Reported AUC values denote the mean ± standard deviation across seeds.

Figure 1 shows the seed-averaged ROC curves for the proposed DMD-CPP method and the DMD-PCA comparison pipeline across the three binary classification tasks. Overall, DMD-CPP yielded higher mean ROC-AUC values than DMD-PCA in all tasks. The strongest performance was observed for FTD vs. CN, where DMD-CPP achieved an AUC of 0.864 ± 0.012, compared with 0.831 ± 0.022 for DMD-PCA. This result indicates that the proposed clustering-based projection was particularly effective in separating FTD subjects from CN controls under eyes-closed resting-state conditions.

For AD vs. CN, DMD-CPP achieved an AUC of 0.795 ± 0.006, which was slightly higher than the corresponding DMD-PCA result of 0.789 ± 0.016. Although the absolute difference was modest, the lower standard deviation of DMD-CPP suggests more stable performance across repeated nested LOSO-CV runs. In contrast, AD vs. FTD remained the most difficult task for both methods. DMD-CPP achieved an AUC of 0.622 ± 0.014, whereas DMD-PCA achieved an AUC of 0.580 ± 0.038. Thus, although discrimination between the two dementia groups was limited, DMD-CPP still provided a consistent improvement over the PCA-based representation.

These ROC-AUC results support two main observations. First, the proposed CPP representation improved threshold-independent discriminative performance relative to PCA-based projection, suggesting that class-specific medoid dictionaries captured information not fully preserved by global variance-based dimensionality reduction. Second, the task-dependent performance pattern was consistent with the clinical and neurophysiological difficulty of the classification problems: disease–control classification was more separable than AD–FTD classification, where overlapping pathological and electrophysiological characteristics may limit subject-level separability.

### 3.2 Subject-level classification performance under LOSO-CV

Table 7 summarizes the subject-level performance obtained under repeated nested LOSO-CV. In this table, balanced accuracy, raw precision, macro-F1, and MCC are reported to evaluate classification performance under class imbalance and to provide a more complete summary of threshold-based subject-level decisions. Consistent with the ROC-AUC results, DMD-CPP outperformed DMD-PCA across all three binary tasks.

**Table 7:** Subject-level performance under LOSO-CV validation.

Method	Task	Balanced acc. ±std	Precision ±std	Macro F1 ±std	MCC ±std
DMD-CPP	AD ± CN	0.7677 ± 0.0253	0.8090 ± 0.0423	0.7653 ± 0.0231	0.5340 ± 0.0510
	FTD ± CN	0.8515 ± 0.0312	0.8422 ± 0.0296	0.8519 ± 0.0301	0.7052 ± 0.0296
	AD ± FTD	0.6382 ± 0.0202	0.7071 ± 0.0130	0.6407 ± 0.0211	0.2900 ± 0.0458
DMD-PCA	AD ± CN	0.7282 ± 0.0263	0.7637 ± 0.0253	0.7272 ± 0.0264	0.4557 ± 0.0529
	FTD ± CN	0.8081 ± 0.0400	0.8053 ± 0.0405	0.8090 ± 0.0379	0.6222 ± 0.0765
	AD ± FTD	0.5574 ± 0.0464	0.6486 ± 0.0303	0.5563 ± 0.0477	0.1238 ± 0.1020

Balanced accuracy, raw precision, macro F1, and Matthews correlation coefficient (MCC) are reported as mean ± standard deviation across repeated nested LOSO-CV runs. Balanced accuracy is computed on decided subjects only.

For AD vs. CN, DMD-CPP achieved a balanced accuracy of 0.7677 ± 0.0253, precision of 0.8090 ± 0.0423, macro-F1 of 0.7653 ± 0.0231, and MCC of 0.5340 ± 0.0510. The corresponding DMD-PCA values were 0.7282 ± 0.0263, 0.7637 ± 0.0253, 0.7272 ± 0.0264, and 0.4557 ± 0.0529, respectively. For FTD vs. CN, DMD-CPP achieved the strongest overall performance among all tasks, with a balanced accuracy of 0.8515 ± 0.0312, precision of 0.8422 ± 0.0296, macro-F1 of 0.8519 ± 0.0301, and MCC of 0.7052 ± 0.0296. In comparison, DMD-PCA achieved 0.8081 ± 0.0400, 0.8053 ± 0.0405, 0.8090 ± 0.0379, and 0.6222 ± 0.0765, respectively. For AD vs. FTD, DMD-CPP again outperformed DMD-PCA, although the absolute performance remained lower than in the disease-control tasks. DMD-CPP yielded a balanced accuracy of 0.6382 ± 0.0202, precision of 0.7071 ± 0.0130, macro-F1 of 0.6407 ± 0.0211, and MCC of 0.2900 ± 0.0458, whereas DMD-PCA yielded 0.5574 ± 0.0464, 0.6486 ± 0.0303, 0.5563 ± 0.0477, and 0.1238 ± 0.1020, respectively.

The improvement of DMD-CPP over DMD-PCA was observed consistently across balanced accuracy, precision, macro-F1, and MCC. This suggests that the proposed class-specific prototype projection improved not only the average balance between sensitivity and specificity, but also the precision of positive diagnostic assignments and the overall balance of the confusion matrix. The improvement was particularly notable in AD vs. FTD, where the MCC increased from 0.1238 for DMD-PCA to 0.2900 for DMD-CPP. This indicates that CPP may provide a more stable decision structure even when the two diagnostic groups share partially overlapping EEG characteristics. However, the relatively low absolute balanced accuracy, macro-F1, and MCC in this task also indicate that AD–FTD discrimination remains substantially more challenging than disease-control classification.

Importantly, these results were obtained under subject-wise validation, in which all epochs from the held-out subject were excluded from both training and model selection. Therefore, the reported performance reflects cross-subject generalization rather than epoch-level memorization. Overall, DMD-CPP showed higher subject-level performance than DMD-PCA across the reported threshold-based metrics, including balanced accuracy, precision, macro-F1, and MCC, although the degree of improvement varied across tasks. The strongest performance was observed in FTD vs. CN, whereas AD vs. CN showed a smaller difference between the two methods and AD vs. FTD remained the most challenging task despite the improvement of DMD-CPP over DMD-PCA.

To evaluate the computational burden of the proposed method, we additionally measured the average runtime of DMD-CPP and DMD-PCA across random seeds for each binary classification task. The results are summarized in Table 8. The reported runtime corresponds to the complete repeated nested LOSO-CV procedure, including inner-loop model selection. Across all tasks, DMD-CPP required longer execution time than DMD-PCA. This indicates that the performance gain achieved by DMD-CPP was accompanied by increased computational cost. The difference is methodologically expected, as DMD-CPP requires class-specific hierarchical clustering and medoid selection during representation learning, whereas DMD-PCA applies a global PCA-based linear projection.

**Table 8:** Average runtime in seconds across random seeds for each classification task and method.

Task	CPP runtime (s)	PCA runtime (s)
AD vs. CN	8,483	532
FTD vs. CN	4,820	256
AD vs. FTD	6,277	514

Runtime was measured for the complete repeated nested LOSO-CV procedure, including inner-loop model selection.

### 3.3 Class-specific medoid patterns learned by DMD-CPP

**Figure 2:**
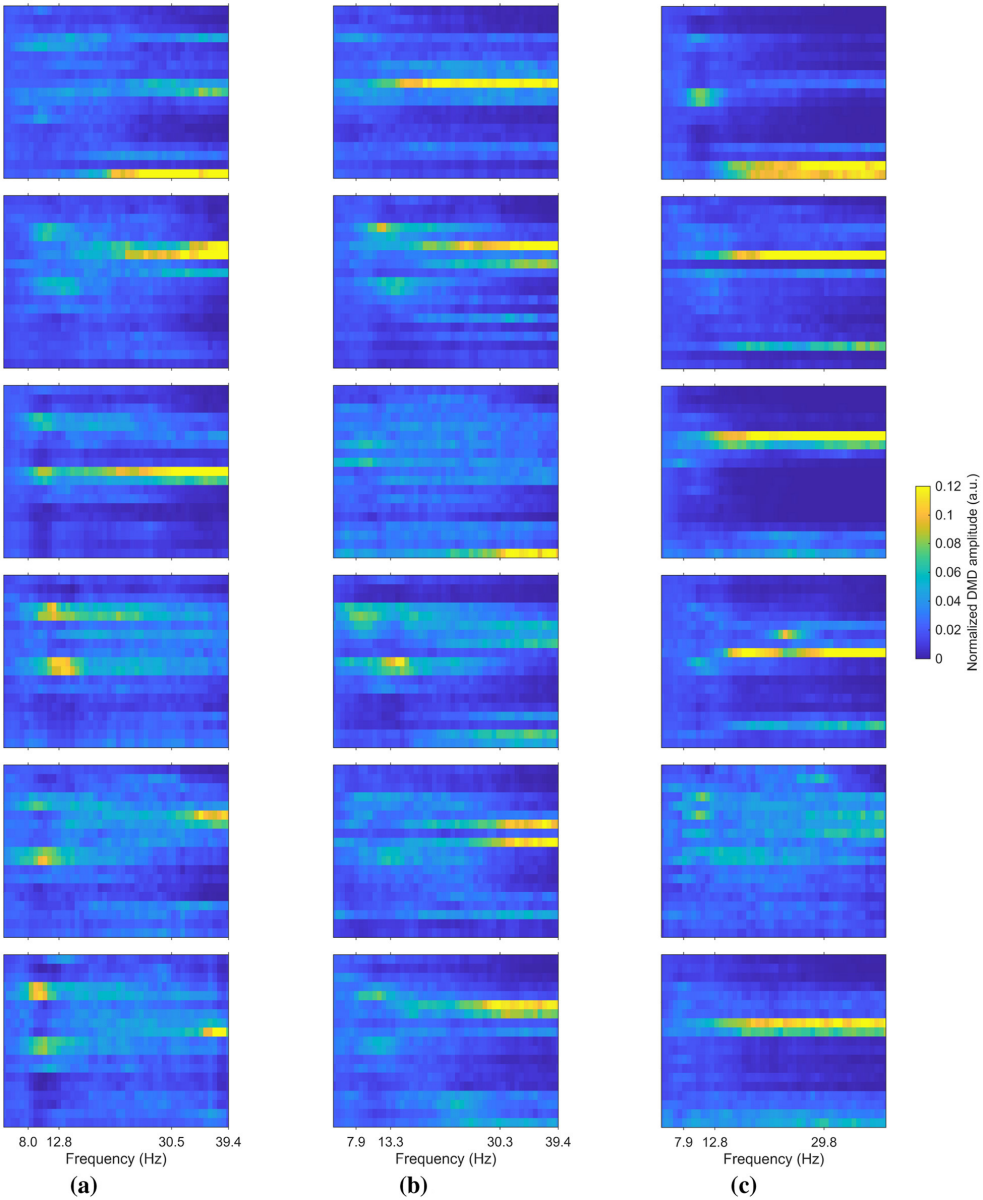
Class-specific medoid learned by DMD-CPP. Shown are up to six representative medoids for each class, selected from the learned class-specific dictionaries. Medoids are ranked using a recursive farthest-first selection procedure. Each medoid is visualized as a channel-by-frequency map derived from epoch-level projected DMD representations. The *x*-axis denotes a resampled DMD frequency axis over retained 4–40 Hz modes. Channels on the *y*-axis are ordered consistently across all columns according to a fixed montage (Pz, Cz, Fz, T6, T5, T4, T3, F8, F7, O2, O1, P4, P3, C4, C3, F4, F3, Fp2, and Fp1). The color scale represents normalized DMD amplitude in arbitrary units (a.u.). **(a)** CN, **(b)** AD, and **(c)** FTD.

Figure 2 visualizes representative class-specific medoids learned by DMD-CPP under the eyes-closed resting-state condition using the most frequently selected CPP hyperparameter configuration across all nested LOSO-CV outer folds and all random seeds. For visualization only, representative medoids were selected from class-specific dictionaries reconstructed from the full dataset using this configuration; these medoids were not used for model selection or test prediction. The maps were generated from unit-normalized DMD basis representations and projected onto a shared class-level frequency axis obtained from the mean interpolated DMD frequency distribution of the selected medoids.

In contrast to our previous eyes-open study, where the pathological groups showed more visibly distorted or frequency-shifted mode structures relative to controls, the present eyes-closed medoids showed broadly comparable frequency ranges across CN, AD, and FTD. In particular, all three groups contained visible low-frequency dynamics within the retained 4–40 Hz range. This suggests that, under resting-state eyes-closed conditions, group differences were not primarily expressed as a complete loss of low-frequency mode structure, but rather as differences in the spatial-frequency organization and recurrence of the learned prototype patterns.

The first two medoids in each class tended to reflect relatively local patterns selected under stricter clustering conditions. They were therefore not interpreted as the main source of global class-level structure. Instead, the latter representative medoids showed more comparable and recurrent configurations across each class-specific dictionary. These medoids are more relevant for interpreting the global prototype structure captured by CPP. In this view, the CN medoids were characterized by relatively short high-frequency components and more complex low-frequency dynamics, whereas the dementia groups showed more extended high-frequency structures.

Among the two dementia groups, AD and FTD showed different forms of disorder-related organization. The AD medoids exhibited weaker low-frequency structure than the FTD medoids, although the patterns appeared relatively stable. This may explain why AD-related resting-state patterns can remain classifiable in conventional disease–control settings, while the specific pattern structure captured by CPP may be less distinctive than in FTD. In contrast, the FTD medoids showed more prominent low-frequency activity around the 8–13 Hz range, corresponding approximately to the alpha band. In addition, one FTD medoid displayed a more fragmented and irregular pattern in the approximately 13–30 Hz range, corresponding broadly to the beta band, and weaker but similar configurations were also observed in other FTD medoids.

A qualitative comparison with previous feature-selection results provides additional context for interpreting these medoids. Ma et al. (2024b) reported that EEG-based dementia classification can depend strongly on selected electrode-pair communication features, and that feature refinement improved AD–FTD discrimination by removing features unnecessary for subtype separation. In their analysis, AD-related discrimination was mainly associated with frontal or frontal–temporal electrode-pair features, whereas FTD-related discrimination involved more temporal–parietal and posterior electrode-pair features. Although the present DMD-CPP medoids are not pairwise communication measures, the fixed channel order allows a qualitative topographic comparison. In the AD medoids, repeated band-like activations were partly observed in frontal-related rows, whereas the FTD medoids showed more irregular and recurrent activations around temporal, parietal, and posterior channel regions, including rows corresponding to T6, T5, T4, T3, P4, P3, O2, and O1. This correspondence should not be interpreted as direct equivalence between the two feature types. Rather, it supports the broader view that dementia-related EEG information may be concentrated in selected spatial or spatial-frequency subspaces rather than expressed as a globally uniform abnormality.

These observations are consistent with the main hypothesis of this study. Under eyes-closed resting-state EEG, the discriminative structure captured by CPP does not necessarily correspond to a simple frequency-band shift or to a uniformly preserved disease biomarker. Instead, CPP appears to identify recurrent class-specific prototype configurations, including fragmented or weakly organized patterns. The stronger performance of DMD-CPP in the FTD vs. CN task, therefore, suggests that such disorder-like patterns can serve as anchors for subject-level classification when they recur across epochs and subjects.

### 3.4 Subject-level decision-margin distributions

**Figure 3:**
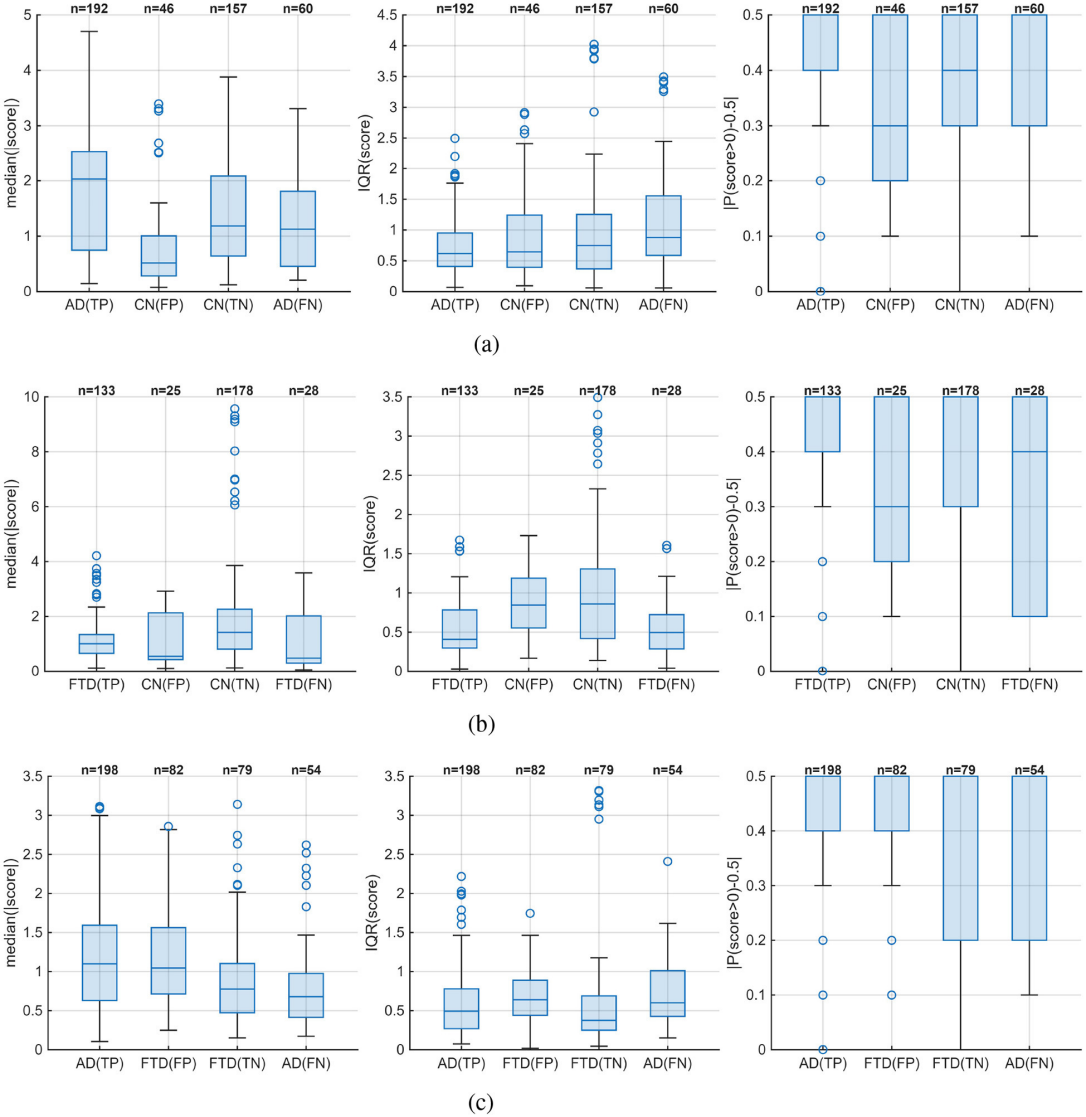
Subject-level decision-margin distributions. Boxplots depict subject-level score descriptors grouped by true → predicted outcome categories, aggregated across all seeds. For each task, panels show (left) the median absolute margin median(|*m*|), (middle) within-subject dispersion IQR(*m*), and (right) sign-consistency |*P*(*m*>0)−0.5|. Tasks shown are **(a)** AD vs. CN, **(b)** FTD vs. CN, and **(c)** AD vs. FTD. Each box summarizes the distribution across subjects pooled over all seeds within the corresponding outcome group (TP, FN, TN, and FP). The reported *n* values correspond to seed-specific subject-level evaluation instances accumulated across repeated random-seed runs, rather than fully independent biological subjects.

Figure 3 shows the distributions of three subject-level decision-margin descriptors for each classification task: the median absolute margin median(|*m*|), within-subject margin dispersion IQR(*m*), and sign-consistency |*P*(*m*>0)−0.5|. For visualization, the boxplots aggregate the seed-specific subject-level evaluation instances across all repeated random-seed runs. The corresponding numerical summaries are provided in Table 9. To avoid treating repeated seed-specific evaluations as independent biological subjects, the statistics in Table 9 were computed separately for each random seed and then summarized across seeds as mean ± standard deviation. For each seed, confidence intervals were computed as two-sided 95% bootstrap confidence intervals using percentile resampling; the reported intervals correspond to the across-seed average of these seed-specific intervals.

**Table 9:** Subject-level score distribution statistics under repeated LOSO validation.

Task	Group	Median(|m|)	IQR (m)	|*P*(*m*>0)−0.5|
AD vs. CN	AD-correct (TP)	1.813 ± 1.050 [0.747,2.524]	0.716 ± 0.446 [0.408,0.952]	0.433 ± 0.134 [0.400,0.500]
	CN-miss (FP)	0.898 ± 0.972 [0.283,1.005]	0.936 ± 0.801 [0.394,1.242]	0.285 ± 0.152 [0.200,0.500]
	CN-correct (TN)	1.441 ± 0.968 [0.643,2.086]	0.898 ± 0.779 [0.369,1.254]	0.368 ± 0.165 [0.300,0.500]
	AD-miss (FN)	1.272 ± 0.896 [0.455,1.809]	1.143 ± 0.878 [0.588,1.554]	0.392 ± 0.148 [0.300,0.500]
FTD vs. CN	FTD-correct (TP)	1.153 ± 0.789 [0.656,1.342]	0.533 ± 0.356 [0.297,0.784]	0.425 ± 0.149 [0.400,0.500]
	CN-miss (FP)	1.049 ± 0.886 [0.431,2.130]	0.873 ± 0.513 [0.553,1.189]	0.352 ± 0.158 [0.200,0.500]
	CN-correct (TN)	1.898 ± 1.744 [0.811,2.264]	1.005 ± 0.722 [0.419,1.306]	0.408 ± 0.155 [0.300,0.500]
	FTD-miss (FN)	1.126 ± 1.274 [0.300,2.023]	0.584 ± 0.413 [0.287,0.724]	0.343 ± 0.175 [0.100,0.500]
AD vs. FTD	AD-correct (TP)	1.190 ± 0.725 [0.630,1.593]	0.601 ± 0.422 [0.269,0.779]	0.423 ± 0.141 [0.400,0.500]
	FTD-miss (FP)	1.181 ± 0.613 [0.714,1.562]	0.665 ± 0.382 [0.440,0.889]	0.438 ± 0.123 [0.400,0.500]
	FTD-correct (TN)	0.908 ± 0.640 [0.472,1.103]	0.665 ± 0.835 [0.250,0.689]	0.380 ± 0.171 [0.200,0.500]
	AD-miss (FN)	0.833 ± 0.611 [0.415,0.975]	0.764 ± 0.523 [0.427,1.012]	0.385 ± 0.139 [0.200,0.500]

For each random seed, margin descriptors were computed separately within each outcome group. Values are reported as the mean ± standard deviation of the seed-specific statistics across repeated random-seed runs. Confidence intervals were computed separately within each seed and are reported as the across-seed average interval. Each subject is assigned to one of four outcome groups based on majority-vote prediction: true positive (TP), false positive (FP), true negative (TN), and false negative (FN).

For AD vs. CN, the margin summaries showed the clearest separation in the median absolute margin. AD-correct subjects tended to show larger median(|*m*|) than CN subjects misclassified as AD, indicating that correctly classified AD cases were generally associated with stronger decision margins than false-positive CN cases. The remaining margin descriptors showed more overlap across outcome groups, although sign-consistency was generally higher for correctly classified subjects than for false-positive cases.

For FTD vs. CN, the group differences were more evident in the within-subject dispersion descriptor IQR(*m*) than in median absolute margin alone. In particular, FTD-correct subjects showed lower within-subject margin dispersion than CN-related outcome groups, whereas CN-correct and CN-miss cases tended to show broader margin variability. This indicates that the FTD vs. CN task was not characterized only by uniformly large margins, but also by task-dependent differences in the stability of epoch-level decision scores within each subject.

For AD vs. FTD, the three margin descriptors showed greater overlap across the four outcome categories than in the two disease-vs.-control tasks. Although DMD-CPP improved classification performance relative to DMD-PCA in this task, the margin distributions indicate that the subject-level decision geometry was less clearly separated. This is consistent with the lower absolute AD vs. FTD performance and supports the interpretation that direct subtype discrimination remains substantially more difficult than disease-vs.-control classification.

Overall, the margin-distribution results show that the descriptor most clearly separating outcome groups differed by task. In AD vs. CN, the most prominent separation appeared in the median absolute margin between TP and FP groups. In FTD vs. CN, group differences were more visible in within-subject margin dispersion. In AD vs. FTD, the margin descriptors showed greater overlap across outcome groups. These findings support a cautious interpretation of the medoid-based disorder-pattern analysis: weak or fragmented DMD-derived patterns were considered meaningful only when they were accompanied by reproducible subject-level margin structure under repeated LOSO-CV, rather than by visual medoid appearance alone.

### 3.5 Permutation-based Decision-Margin Analysis

Table 10 reports the permutation-based group-difference analysis of subject-level margin descriptors for the proposed DMD-CPP method. To avoid treating repeated random-seed evaluations as independent biological samples, all statistics were first computed separately within each seed and then summarized across seeds.The table reports the mean and standard deviation of the seed-specific median differences Δmed, the across-seed average of the 95% bootstrap confidence intervals, the median permutation *p*-value across seeds with its minimum and maximum, and the corresponding Cliff's δ summaries. Therefore, the reported *p*-values should be interpreted as summaries of seed-specific statistical evidence rather than as a single pooled-subject permutation result.

**Table 10:** Permutation-based group-difference analysis of subject-level margin descriptors using seed-wise summaries.

Task	Descriptor	Compare	Δmed ±std [95% CI]	*p*_med_ [min, max]	Cliff's δ ±std [95% CI]	*p*_δ, med_ [min, max]
AD vs. CN	median(|*m*|)	(TP–FP)	1.480 ± 0.236 [–0.159,2.011]	0.0364 [0.0016,0.2072]	0.474 ± 0.135 [–0.095,0.895]	0.0382 [0.0083,0.5129]
		(TN–FN)	0.076 ± 0.263 [–0.993,1.057]	0.9108 [0.2655,0.9580]	0.093 ± 0.130 [–0.379,0.547]	0.7107 [0.2205,0.9322]
	IQR(*m*)	(TP–FP)	–0.005 ± 0.124 [–0.837,0.350]	0.6350 [0.3396,1.0000]	–0.045 ± 0.168 [–0.590,0.486]	0.7046 [0.2347,0.8109]
		(TN–FN)	–0.217 ± 0.229 [–1.048,0.415]	0.6495 [0.1208,0.9734]	–0.187 ± 0.089 [–0.640,0.300]	0.3478 [0.2546,0.9154]
	|*P*(*m*>0)−−0.5|	(TP–FP)	0.200 ± 0.112 [0.071,0.357]	0.0636 [0.0104,1.0000]	0.469 ± 0.206 [0.073,0.855]	0.0108 [0.0009,0.8717]
		(TN–FN)	–0.036 ± 0.075 [–0.157,0.214]	0.6892 [0.3838,1.0000]	–0.096 ± 0.190 [–0.487,0.330]	0.3306 [0.1626,0.8550]
FTD vs. CN	median(|*m*|)	(TP–FP)	0.317 ± 0.367 [–1.511,0.859]	0.2666 [0.1373,0.6922]	0.145 ± 0.285 [–0.663,0.797]	0.4112 [0.2276,0.8101]
		(TN–FN)	0.732 ± 0.489 [–1.826,1.607]	0.1647 [0.1269,0.7258]	0.418 ± 0.163 [–0.460,0.988]	0.1226 [0.0502,0.9015]
	IQR(*m*)	(TP–FP)	–0.361 ± 0.108 [–1.185,0.207]	0.1486 [0.0785,0.5218]	–0.395 ± 0.182 [–0.952,0.340]	0.2596 [0.0721,0.7151]
		(TN–FN)	0.322 ± 0.181 [–0.243,0.735]	0.3449 [0.1733,0.9631]	0.395 ± 0.224 [–0.091,0.819]	0.3831 [0.0416,0.7561]
	|*P*(*m*>0)−−0.5|	(TP–FP)	0.107 ± 0.110 [–0.029,0.329]	0.1444 [0.0437,1.0000]	0.225 ± 0.210 [–0.285,0.781]	0.3757 [0.0593,1.0000]
		(TN–FN)	0.143 ± 0.140 [0.000,0.343]	0.2823 [0.0093,1.0000]	0.208 ± 0.200 [–0.314,0.743]	0.6577 [0.0196,1.0000]
AD vs. FTD	median(|*m*|)	(TP–FP)	0.016 ± 0.186 [–0.700,0.485]	0.7486 [0.3967,0.8872]	–0.020 ± 0.111 [–0.412,0.368]	0.8162 [0.3208,0.9887]
		(TN–FN)	0.062 ± 0.213 [–0.646,0.630]	0.6679 [0.0204,0.9770]	0.068 ± 0.232 [–0.464,0.598]	0.6049 [0.0697,1.0000]
	IQR(*m*)	(TP–FP)	–0.172 ± 0.104 [–0.434,0.171]	0.2818 [0.0594,0.9001]	–0.151 ± 0.102 [–0.546,0.259]	0.3559 [0.2123,0.9648]
		(TN–FN)	–0.210 ± 0.156 [–0.850,0.207]	0.3657 [0.0425,0.9042]	–0.314 ± 0.168 [–0.787,0.218]	0.4163 [0.0331,0.5989]
	|*P*(*m*>0)−0.5|	(TP–FP)	0.000 ± 0.000 [–0.007,0.071]	1.0000 [1.0000,1.0000]	–0.031 ± 0.095 [–0.319,0.280]	0.6297 [0.3490,1.0000]
		(TN–FN)	0.029 ± 0.049 [–0.264,0.286]	1.0000 [0.4227,1.0000]	0.017 ± 0.149 [–0.438,0.495]	0.5998 [0.4084,0.9407]

For each random seed, group differences were computed separately for the predicted-positive group (TP vs. FP) and the predicted-negative group (TN vs. FN). The table reports the mean ± standard deviation of seed-specific median differences, Δmed, the across-seed average of the 95% bootstrap confidence intervals (CI), the median permutation *p*-value across seeds (*p*_med_; 20,000 permutations with fixed group sizes) with its minimum and maximum, and the mean ± standard deviation of seed-specific Cliff's δ with the corresponding averaged confidence intervals and median permutation *p*-values.

For AD vs. CN, the clearest and most consistent separation was observed in the predicted-positive comparison for the median absolute margin. The TP–FP difference in median(|*m*|) was positive across seeds, with Δmed = 1.480 ± 0.236, and the median permutation test was significant (*p*_med_ = 0.0364). Cliff's δ showed a similar pattern, with δ = 0.474 ± 0.135 and *p*_δ, med_ = 0.0382. This indicates that correctly classified AD subjects tended to have stronger decision margins than CN subjects misclassified as AD. Sign-consistency also showed a positive TP–FP tendency, but the median-difference test was only borderline (*p*_med_ = 0.0636), whereas the Cliff's δ test was significant in the median across seeds (*p*_δ, med_ = 0.0108). The TN–FN comparisons did not show stable statistical support across descriptors.

For FTD vs. CN, several descriptors showed task-relevant effect directions, but the seed-wise permutation evidence was less stable. In the predicted-positive comparison, IQR(*m*) showed a negative TP–FP difference (Δmed = −0.361 ± 0.108), indicating lower within-subject margin dispersion in correctly classified FTD subjects than in CN subjects misclassified as FTD. However, the median permutation *p*-value was not significant (*p*_med_ = 0.1486), and the seed-wise *p*-value range indicated variability across seeds. In the predicted-negative comparison, both median(|*m*|) and IQR(*m*) showed positive differences, but their median permutation *p*-values were also not significant. Thus, compared with AD vs. CN, the FTD vs. CN task showed meaningful directional margin patterns, particularly in within-subject dispersion, but the statistical evidence varied across seeds. This variability may reflect the smaller number of FTD subjects and the sensitivity of the learned CPP dictionary to the seed-specific inner-loop selection process.

For AD vs. FTD, the permutation results showed no robust group separation in median absolute margin or sign-consistency. The TP–FP and TN–FN comparisons for median(|*m*|) had small mean differences and non-significant median permutation *p*-values. The IQR(*m*) descriptor showed negative mean differences for both TP–FP and TN–FN comparisons, suggesting a tendency toward lower dispersion in correctly classified groups, but the median permutation *p*-values were not significant. These results indicate that the AD vs. FTD margin structure was weaker and less stable than in the disease-vs.-control tasks, consistent with the lower classification performance observed for this task.

Overall, the seed-wise permutation analysis supports a more cautious interpretation than a pooled-subject analysis. AD vs. CN showed the most stable statistical evidence, mainly in the TP–FP median absolute margin. FTD vs. CN showed interpretable task-dependent margin trends, especially in within-subject dispersion, but the corresponding *p*-values varied substantially across seeds. AD vs. FTD showed limited and unstable margin separation. These findings suggest that the reliability of CPP-derived disorder-like patterns should be assessed not only by the direction of the margin differences, but also by their stability across repeated seed-specific model-selection runs.

Table 11 compares the proposed DMD-CPP representation with the DMD-PCA baseline using the same subject-level margin descriptors. The comparison was restricted to the predicted-positive group, namely TP–FP, in order to evaluate whether correctly classified positive-class subjects showed stronger or more reliable margin structure than negative-class subjects misclassified as positive. To avoid treating repeated random-seed evaluations as independent biological samples, all statistics were first computed separately within each seed and then summarized across seeds. The table reports the mean and standard deviation of the seed-specific median differences Δmed, averaged 95% bootstrap confidence intervals, Cliff's δ, and the median permutation *p*-value across seeds with its minimum and maximum.

**Table 11:** Permutation-based evaluation of subject-level margin descriptors under repeated LOSO validation.

Task	Model	median (|m|)			IQR (m)		
		Δmed ±std [95% CI]	Cliff's δ ±std [95% CI]	*p*_δ, med_ [min, max]	Δmed ±std [95% CI]	Cliff's δ ±std [95% CI]	*p*_δ, med_ [min, max]
AD vs. CN	DMD-CPP	1.480 ± 0.236 [–0.159,2.011]	0.474 ± 0.135 [–0.095,0.895]	0.0382 [0.0083,0.5129]	–0.005 ± 0.124 [–0.837,0.350]	–0.045 ± 0.168 [–0.590,0.486]	0.7047 [0.2347,0.8109]
	DMD-PCA	1.167 ± 0.329 [0.035,1.679]	0.466 ± 0.134 [0.077,0.718]	0.0402 [0.0028,0.3470]	–0.101 ± 0.169 [–0.624,0.390]	–0.044 ± 0.220 [–0.477,0.264]	0.3858 [0.1311,0.9202]
FTD vs. CN	DMD-CPP	0.317 ± 0.367 [–1.511,0.859]	0.145 ± 0.285 [–0.663,0.797]	0.4112 [0.2275,0.8101]	–0.361 ± 0.108 [–1.185,0.207]	–0.395 ± 0.182 [–0.952,0.340]	0.2597 [0.0720,0.7151]
	DMD-PCA	1.030 ± 0.835 [–0.552,3.009]	0.337 ± 0.233 [0.037,0.820]	0.2934 [0.0398,0.9576]	0.665 ± 0.838 [–2.168,2.052]	0.064 ± 0.270 [–0.259,0.714]	0.4490 [0.2945,0.9628]
AD vs. FTD	DMD-CPP	0.016 ± 0.186 [–0.700,0.485]	–0.020 ± 0.111 [–0.412,0.368]	0.8162 [0.3208,0.9887]	–0.172 ± 0.104 [–0.434,0.171]	–0.151 ± 0.102 [–0.546,0.259]	0.3559 [0.2123,0.9648]
	DMD-PCA	0.420 ± 0.593 [–0.740,1.930]	0.125 ± 0.109 [–0.152,0.455]	0.4715 [0.1320,0.8295]	0.264 ± 0.260 [–0.502,0.999]	0.077 ± 0.108 [–0.176,0.464]	0.6109 [0.2514,0.9429]

For each task and model, TP–FP differences were evaluated within the predicted-positive group. Δmed and Cliff's δ were computed separately for each seed and reported as mean ± standard deviation across seeds. Bracketed values indicate the across-seed average 95% bootstrap confidence intervals. Permutation *p*-values are reported as median values with minimum and maximum across seeds.

For AD vs. CN, both DMD-CPP and DMD-PCA showed a consistent TP–FP separation in median absolute margin. DMD-CPP yielded Δmed = 1.480 ± 0.236, Cliff's δ = 0.474 ± 0.135, and a median permutation *p*-value of 0.0382. DMD-PCA showed a similar effect, with Δmed = 1.167 ± 0.329, δ = 0.466 ± 0.134, and a median permutation *p*-value of 0.0402. Thus, both representations indicated that correctly classified AD subjects tended to have stronger decision margins than CN subjects misclassified as AD. The median-margin difference was numerically larger for DMD-CPP, whereas the effect-size estimates were comparable. In contrast, neither method showed a stable TP–FP effect in IQR(*m*), indicating that the main reliability-related contrast in AD vs. CN was associated with margin magnitude rather than within-subject dispersion.

For FTD vs. CN, the TP–FP effects were less stable across seeds. DMD-CPP showed a negative difference in IQR(*m*), with Δmed = −0.361 ± 0.108 and δ = −0.395 ± 0.182, suggesting lower within-subject margin dispersion in correctly classified FTD subjects than in CN subjects misclassified as FTD. However, the median permutation *p*-value was not significant (*p*_δ, med_ = 0.2597), and the seed-wise range indicated variability across random-seed runs. DMD-PCA showed a larger positive median-margin difference (Δmed = 1.030 ± 0.835), but this effect also showed high seed-to-seed variability and did not reach significance in the median permutation summary (*p*_δ, med_ = 0.2934). These results suggest that FTD vs. CN contained meaningful margin trends. Still, the specific descriptor showing stronger separation depended on the representation and was sensitive to seed-specific model selection, likely reflecting the smaller FTD sample size.

For AD vs. FTD, neither method showed a robust TP–FP separation in either median absolute margin or IQR(*m*). DMD-CPP showed near-zero median-margin difference (Δmed = 0.016 ± 0.186) and a non-significant effect size summary (*p*_δ, med_ = 0.8162). DMD-PCA showed a larger numerical median-margin difference (Δmed = 0.420 ± 0.593), but the variability was large, and the median permutation summary was not significant. For IQR(*m*), both methods also showed non-significant TP–FP effects. These findings indicate that AD vs. FTD did not produce a reliable positive-class margin contrast, consistent with the lower absolute classification performance for this task.

Overall, Table 11 shows that the reliability-related margin structure differed across tasks and methods. AD vs. CN showed the most stable TP–FP separation in median absolute margin for both DMD-CPP and DMD-PCA. FTD vs. CN showed task-relevant but seed-sensitive margin trends, with DMD-CPP tending to reflect differences in within-subject dispersion and DMD-PCA tending to reflect differences in median margin magnitude. AD vs. FTD showed no robust TP–FP margin separation for either representation. These results support a cautious interpretation: DMD-derived representations can reveal margin structures beyond accuracy alone, but the stability of these structures depends on the task, the representation, and the seed-specific model-selection process.

## 4 Discussion

A central question of this study was whether EEG patterns that appear weak, fragmented, or unstable can still form recurrent subject-level discriminative structure, particularly in disease–control settings. Conventional EEG-based dementia classification often assumes that diagnostically useful information is expressed through relatively stable biomarkers, such as spectral slowing, altered complexity, or disrupted functional connectivity. This assumption is well motivated in many eyes-closed AD studies, where resting-state abnormalities have been repeatedly reported and can be summarized using spectral, entropy-based, connectivity, or covariance-based representations (Cassani et al., 2018; Jeong, 2004; Rossini et al., 2020). However, the present results suggest that classification-relevant EEG structure need not always appear as a clean or uniformly preserved oscillatory signature.

The strongest support for this view came from the FTD vs. CN task. Under strict nested LOSO-CV, DMD-CPP achieved its highest disease-control performance in FTD vs. CN, even though FTD-related resting-state EEG abnormalities are often described as weaker, less consistent, or more heterogeneous than those observed in AD (Mlinarič et al., 2026; Nishida et al., 2011; Zheng et al., 2025). The medoid patterns further suggested that the FTD class was not characterized only by a single stable low-frequency signature. Instead, FTD medoids showed both alpha-range activity around 8–13 Hz and more irregular beta-range components around 13–30 Hz. These patterns were not identical across medoids, but they reappeared in related forms within the class-specific dictionary. This recurrence is important: if such patterns were merely random noise, they would be unlikely to form reusable medoid anchors that support subject-level classification across held-out subjects.

The decision-margin results provide additional context for this interpretation. In FTD vs. CN, the margin behavior was not explained solely by the magnitude of the margin. In particular, DMD-CPP showed a tendency for correctly classified FTD subjects to have lower within-subject dispersion IQR(*m*) than CN subjects misclassified as FTD. This suggests that the classification-relevant structure may be partly reflected in the stability of epoch-level projections within a subject, rather than only in the absolute strength of the decision margin. Thus, the informative pattern was not necessarily a uniformly strong response across all epochs, but a seed-sensitive subject-level decision tendency involving both stable and variable epoch projections. In this sense, disorder is not equivalent to noise. Noise is idiosyncratic and non-reproducible, whereas a disorder-like pattern may be heterogeneous at the epoch level while still showing recurrent tendencies across subjects in a class-specific manner. However, because the dispersion-related effect was not statistically stable across random seeds, this interpretation should be regarded as suggestive rather than as robust statistical evidence.

This interpretation is consistent with our previous EO study, where AD-related EO responses appeared physiologically degraded, but CPP-based representation learning still improved classification and produced more informative decision-margin structure (Seo et al., 2026). The present EC results extend that observation to a different condition and diagnostic context. In EO, the disorder pattern was associated mainly with degraded AD responses under stimulation; in EC, the present results suggest that FTD-related resting-state dynamics may also contain recurrent disorder-like configurations that can be captured by prototype-based projection. Therefore, the recurrence of disorder-like patterns in this computational setting should not be understood as the preservation of a single canonical waveform or frequency-band abnormality. Rather, it may reflect the recurrence of task-relevant spatial-frequency configurations that remain detectable despite local instability, especially in the FTD vs. CN setting.

At the same time, the AD vs. FTD results show the limitation of this interpretation. Although DMD-CPP improved performance relative to the PCA-based comparison, both the ROC-AUC and margin analyses indicated that AD vs. FTD remained substantially less separable than the disease-control tasks. This agrees with prior work suggesting that AD and FTD share partially overlapping clinical and electrophysiological features, which can limit direct subtype discrimination (Mlinarič et al., 2026; Ma et al., 2024b; Pérez-Millan et al., 2024). Thus, disorder-like patterns may support subject-level discrimination when they provide a recurring contrast against cognitively normal controls, but they may be insufficient for reliable differential classification when two pathological groups share overlapping or interacting forms of network disruption.

Taken together, these findings support a limited interpretation: unstable-looking EEG responses can contain recurrent structure that contributes to subject-level discrimination, particularly in disease-control settings. The key requirement is not that every epoch exhibits a clean and stable biomarker, but that the class produces recurrent prototype configurations that are sufficiently consistent to be learned from training subjects and recognized in held-out subjects. DMD-CPP provides one way to expose this structure by converting heterogeneous DMD mode descriptors into similarity coordinates relative to class-specific medoid dictionaries. From this perspective, disorder patterns should not be treated automatically as irrelevant or purely artifactual; in some dementia EEG settings, they may constitute part of a classification-relevant representation when supported by recurrence and subject-level margin structure.

An additional observation is that the asymmetric task performance was not unique to CPP. The DMD-PCA baseline also showed relatively strong FTD vs. CN performance compared with AD vs. CN and AD vs. FTD. This suggests that the DMD mode descriptors themselves may already contain information related to disorder-like resting-state dynamics. In other words, the ability to capture FTD-related structure may not arise solely from the CPP projection stage; rather, DMD appears to provide a modal representation in which weak, heterogeneous, or fragmented classification-relevant dynamics can become partially measurable.

However, the margin analysis indicates why this observation alone is insufficient. PCA projects the DMD descriptors onto directions that preserve global variance. Therefore, it may capture broad differences between outcome groups without isolating whether those differences correspond to recurrent class-specific decision anchors. Indeed, the PCA baseline also produced several apparent group differences in margin descriptors, but confidence intervals or permutation statistics did not consistently support some of these effects. Therefore, the presence of a numerical margin difference in the PCA representation does not necessarily imply that the learned structure is a recurrent and reliability-supported disorder-like pattern. This is precisely where the present study differs from a conventional performance comparison: we do not interpret classification accuracy alone as exploratory evidence of reliable pattern recurrence, but examine whether the learned representation induces reliable subject-level decision geometry.

From this perspective, DMD and CPP play complementary roles. DMD provides a mode-based representation capable of exposing non-stationary and heterogeneous EEG dynamics, whereas CPP restricts the final feature space to recurrent medoid patterns learned from the training subjects. Thus, CPP does not simply amplify all DMD-derived variability. Instead, it selectively converts repeatedly observed mode configurations into prototype-similarity coordinates. This distinction is important for interpreting disorder patterns. A fragmented or unstable-looking response should not be considered meaningful merely because it improves classification performance; it becomes meaningful only when it recurs across subjects and produces a consistent margin structure under subject-wise validation.

The present EC results also differ from our previous EO findings. In the EO setting, margin-based reliability was most clearly expressed in the AD-related TP–FP contrast, suggesting that CPP captured a degraded but recurring AD-like response pattern (Seo et al., 2026). In the current EC setting, the FTD vs. CN task showed a broader margin organization, particularly through IQR(*m*) differences across outcome groups. This suggests that FTD-related EC structure may not be represented by a single high-confidence disease pattern alone. Rather, it may involve a mixture of relatively preserved resting-state components and disorder-like epoch-level variability. The fact that this variability was reflected in within-subject margin dispersion supports the interpretation that FTD-related disorder-like patterns can contribute to subject-level discrimination under strict validation when they recur in a systematic way.

Accordingly, the main contribution of the present study is not only that DMD-CPP achieved competitive classification performance, but that it provides an analysis framework for examining whether the captured patterns are supported by subject-level reliability evidence. DMD-PCA results indicate that DMD mode features can contain disorder-related information, but CPP and margin analysis make it possible to evaluate whether such information forms recurrent class-specific decision anchors. This reliability-oriented interpretation is essential in small EEG datasets, where even subject-wise validation can still leave ambiguity about whether a classifier has learned a reproducible subject-level decision structure or a less stable dataset-specific regularity.

The improved performance of DMD-CPP should also be interpreted together with its computational cost. The runtime analysis showed that DMD-CPP required substantially longer execution time than DMD-PCA across all binary tasks. This increase is expected because CPP includes class-specific hierarchical clustering, medoid selection, and repeated inner-loop model selection, whereas PCA uses a global variance-preserving linear projection. Therefore, the advantage of DMD-CPP is not computational efficiency, but its ability to construct interpretable class-specific prototype coordinates and to improve subject-level discrimination under strict validation. In applications where runtime is the primary constraint, DMD-PCA may be preferable. In contrast, DMD-CPP may be more appropriate when reliability and interpretability are the main methodological objectives.

Several limitations remain. The dataset size was limited, especially for FTD, and the results should be validated in larger independent cohorts. The present analysis was restricted to binary tasks and should be extended to multi-class dementia classification and uncertainty-aware decision systems. In addition, the medoid and margin analyses provide representation-level evidence rather than direct neurophysiological proof, and the observed disorder-like patterns should be tested across different preprocessing pipelines, recording conditions, and DMD configurations. The computational cost of DMD-CPP is another practical limitation, particularly because the current implementation requires repeated hierarchical clustering and inner-loop model selection. Future work should therefore investigate more efficient CPP implementations, including approximate clustering, reduced hyperparameter grids, parallelized model selection, and memory-efficient medoid selection. Future work should also establish pre-specified margin-based reliability criteria and evaluate whether they can support rejection, uncertainty estimation, or clinically interpretable subject-level screening. Despite these limitations, the present study shows that DMD-CPP combined with margin analysis can move beyond accuracy-based evaluation and assess whether weak or fragmented EEG responses form reproducible representation-level decision structure under strict subject-wise validation.

Another limitation is that this study used the publicly released preprocessed EEG signals. Although this choice ensured consistency with the shared OpenNeuro dataset and avoided introducing an additional study-specific preprocessing pipeline, the extracted DMD modes and CPP medoids may still be influenced by preprocessing steps such as band-pass filtering, ASR, ICA-based artifact attenuation, average referencing, automated bad-channel handling, and differences in usable recording length across subjects. Therefore, the observed disorder-like medoid patterns should not be interpreted as direct neurophysiological proof of disease mechanisms. Rather, they should be understood as representation-level structures whose relevance is supported by recurrence within CPP dictionaries, held-out subject classification, and subject-level margin behavior under LOSO-CV. Future work should examine whether similar medoid and margin structures are preserved when raw EEG data are reprocessed using alternative preprocessing pipelines and validated in independent cohorts.

## 5 Conclusion

This study investigated whether weak, fragmented, or disorder-like EEG responses can form recurrent subject-level discriminative structure, particularly in disease-control dementia classification. Using DMD-based epoch descriptors and CPP under strict nested LOSO-CV, we found that DMD-CPP achieved competitive subject-level performance, particularly for FTD vs. CN classification, and produced task-dependent margin structures that were not fully captured by accuracy alone. The medoid and margin analyses suggest that DMD features can expose disorder-like, classification-relevant resting-state patterns at the representation level, while CPP can convert recurrent class-specific mode configurations into prototype-based representations. In AD vs. CN, reliability was mainly reflected in TP-FP separation of median margin magnitude, whereas in FTD vs. CN, within-subject margin dispersion provided additional evidence of structured variability. Although AD vs. FTD remained substantially more difficult, the results support the more limited view that classification-relevant EEG information need not appear only as stable canonical biomarkers. Recurrent disorder-like patterns, when consistently observed across subjects and epochs, may contribute to subject-level decision structure under strict validation, but the present findings should be interpreted as exploratory rather than as evidence for practical differential diagnosis. Thus, DMD-CPP combined with margin-based analysis provides a computational framework for evaluating both classification performance and reliability of learned EEG representations under subject-wise validation.

## Data Availability

This study analyzed publicly available data from the OpenNeuro repository (dataset ds004504, version 1.0.8). The dataset can be accessed at: https://openneuro.org/datasets/ds004504/versions/1.0.8.
